# QTL × environment interactions underlie adaptive divergence in switchgrass across a large latitudinal gradient

**DOI:** 10.1073/pnas.1821543116

**Published:** 2019-06-10

**Authors:** David B. Lowry, John T. Lovell, Li Zhang, Jason Bonnette, Philip A. Fay, Robert B. Mitchell, John Lloyd-Reilley, Arvid R. Boe, Yanqi Wu, Francis M. Rouquette, Richard L. Wynia, Xiaoyu Weng, Kathrine D. Behrman, Adam Healey, Kerrie Barry, Anna Lipzen, Diane Bauer, Aditi Sharma, Jerry Jenkins, Jeremy Schmutz, Felix B. Fritschi, Thomas E. Juenger

**Affiliations:** ^a^Department of Plant Biology, Michigan State University, East Lansing, MI 48824;; ^b^Great Lakes Bioenergy Research Center, Michigan State University, East Lansing, MI 48824;; ^c^Plant Resilience Institute, Michigan State University, East Lansing, MI 48824;; ^d^Genome Sequencing Center, HudsonAlpha Institute for Biotechnology, Huntsville, AL 35806;; ^e^Department of Integrative Biology, The University of Texas at Austin, Austin, TX 78705;; ^f^Grassland, Soil and Water Research Laboratory, Agricultural Research Service, US Department of Agriculture, Temple, TX 76502;; ^g^Wheat, Sorghum, and Forage Research Unit, Agricultural Research Service, US Department of Agriculture, University of Nebraska–Lincoln, Lincoln, NE 68583;; ^h^Kika de la Garza Plant Materials Center, National Resources Conservation Service, US Department of Agriculture, Kingsville, TX 78363;; ^i^Department of Agronomy, Horticulture & Plant Science, South Dakota State University, Brookings, SD 57007;; ^j^Department of Plant and Soil Sciences, Oklahoma State University, Stillwater, OK 74075;; ^k^Texas A&M AgriLife Research, Texas A&M AgriLife Research and Extension Center, Texas A&M University, Overton, TX 75684;; ^l^Plant Materials Center, National Resources Conservation Service, US Department of Agriculture, Manhattan, KS 66502;; ^m^Department of Energy Joint Genome Institute, Walnut Creek, CA 94598;; ^n^Division of Plant Sciences, University of Missouri, Columbia, MO 65201

**Keywords:** bioenergy, ecotype, local adaptation, plasticity, G × E

## Abstract

Local adaptation is the process by which natural selection drives adaptive phenotypic divergence across environmental gradients. Theory suggests that local adaptation results from genetic trade-offs at individual genetic loci, where adaptation to one set of environmental conditions results in a cost to fitness in alternative environments. However, the degree to which there are costs associated with local adaptation is poorly understood because most of these experiments rely on two-site reciprocal transplant experiments. Here, we quantify the benefits and costs of locally adaptive loci across 17° of latitude in a four-grandparent outbred mapping population in outcrossing switchgrass (*Panicum virgatum* L.), an emerging biofuel crop and dominant tallgrass species. We conducted quantitative trait locus (QTL) mapping across 10 sites, ranging from Texas to South Dakota. This analysis revealed that beneficial biomass (fitness) QTL generally incur minimal costs when transplanted to other field sites distributed over a large climatic gradient over the 2 y of our study. Therefore, locally advantageous alleles could potentially be combined across multiple loci through breeding to create high-yielding regionally adapted cultivars.

## Significance

Understanding how individual genetic loci contribute to trait variation across geographic space is of fundamental importance for understanding evolutionary adaptations. Our study demonstrates that most loci underlying locally adaptive trait variation have beneficial effects in some geographic regions while conferring little or no detectable cost in other parts of the geographic range of switchgrass over two field seasons of study. Thus, loci that contribute to local adaptation vary in the degree to which they are costly in alternative environments but typically confer greater benefits than costs. Further, our study suggests that breeding locally adapted varieties of switchgrass will be a boon to the biofuel industry, as locally adaptive loci could be combined to increase local yields in switchgrass.

Local adaptation is one of the major drivers of biodiversity, as variable natural selection along environmental gradients increases phenotypic and genetic diversity within species and provides the raw material for speciation (1–4). Despite the importance of local adaptation, we have a poor understanding its genetic basis, especially concerning how individual genetic loci contribute to adaptation across environmental gradients (4, 5). Theoretical models predict that local adaptation should involve strong fitness trade-offs (i.e., antagonistic pleiotropy) at the level of individual loci (6–9). Well-known studies of adaptation, including the evolution of beak size in Darwin’s finches (10), coat color of mice (11, 12), and flower morphology in monkeyflowers (13), also appear to support the importance of strong trade-offs in local adaptation. However, studies that have combined reciprocal transplant field experiments with quantitative trait locus (QTL) mapping (14–19) and genome-wide association studies (20) have found that trade-offs at the individual locus level are relatively rare [only ∼18% of QTL had detectable fitness trade-offs; reviewed in Wadgymar et al. (5)]. In contrast, loci that have effects on fitness in one environment, but not in alternative environments (i.e., conditional neutrality), appear to be more common (4, 5). While results from these previous genetic studies of local adaptation in the field have advanced our understanding of local adaptation, they have not resolved how often and to what extent loci confer benefits and costs across geographic space.

Previous genetic studies of local adaptation in the field have been restricted in their generalizability for multiple reasons (5). Many of these studies were of short duration or focused on a limited environmental range. As a consequence, these studies cannot rule out the possibility that trade-offs were undetected because of insufficient sample sizes, inadequately sampled environmental conditions, or environmental variability among years (21, 22). These studies have also been primarily restricted to biparental crosses in annual plant species that are predominantly self-fertilizing. The low outcrossing rates and/or patchy distributions of these species could provide mechanisms for the evolution of locally adaptive alleles that have positive effects in one population without spreading to other populations by gene flow (4, 5, 23). Further, experiments to date have primarily relied only on two field sites, often at the extreme ends of environmental gradients (5). Without finer-scale analyses of genetic effects across geographic space, it is not possible to determine how the fitness contributions of individual loci change across environmental gradients. Studies that expand the genetics of local adaptation research to more than two field sites, to outbreeding perennial species, and with crosses involving more parents have the potential to clarify the extent to which there are benefits and cost of locally adaptive loci.

**Fig. 1.**
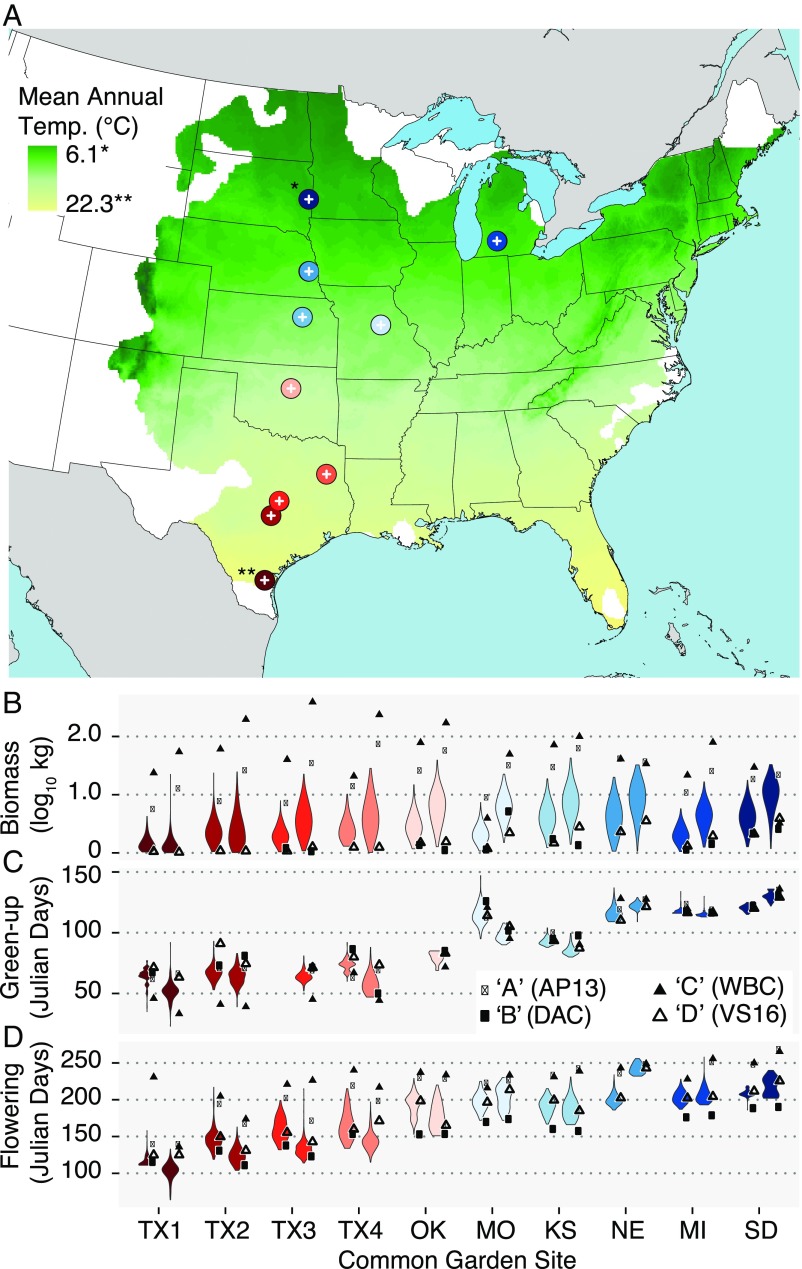
Geographic and environmental variation across 10 common garden sites. (*A*). The 10 common gardens cover 1,866 km, 16.7° of latitude and 16.2 °C of mean annual temperature variation. The latitudinal transect of this study spans much of the natural distribution of switchgrass. The green/yellow layer is colored by historical annual temperature and is bounded by the US distribution of native switchgrass populations, calculated from georeferenced herbarium records. (*B*–*D*) The genotypic means of each of the two southern lowland (AP13, WBC) and two northern upland (VS16, DAC) grandparents as points. The phenotypic distributions of the F_2_ mapping population for three key traits are depicted as violin plots. Data from 2016 (left violin) and 2017 (right violin) are included for each site.

In this study, we expand the scope of local adaptation research by evaluating its genetic basis in outcrossing perennial switchgrass (*Panicum virgatum* L.) across 10 field sites, covering 17° of latitude (1,866 km) in the central United States (Fig. 1). The mapping population used in this study combined the genetic variation of three switchgrass cultivars and one wild accession. Switchgrass cultivars are derived from natural populations and unlike most crop species are only a few generations removed from those wild collections (24). Clones of the same outbred four-way genetic mapping population were planted at each site, which allowed us to evaluate the contributions of individual loci to traits and fitness over a wide range of climatic conditions. The grandparents of the mapping population were derived from highly divergent southern lowland and northern upland ecotypes (25). The southern lowland ecotype of switchgrass is typically found in riparian areas of the southern United States, produces large amounts of biomass, and is more nutrient-use-efficient, heat-tolerant, pathogen-resistant, and flooding-tolerant than the northern upland ecotype (26–30). However, the northern upland ecotype is typically more freezing-tolerant than the southern lowland ecotype (31–35). Flowering time in switchgrass, a trait correlated with biomass production, follows a strong latitudinal pattern, where flowering time becomes progressively later in more southern populations (29, 36–38).

For switchgrass, which is a long-lived perennial grass, biomass is both indicative of potential utility as a bioenergy crop and is an excellent proxy for fitness. Across two field experiments performed by Palik et al. (39), there was a high correlation between dry biomass and the total number of seeds per plant (*R*^2^ = 0.83, both experiments). Clarifying how different genetic loci contribute to biomass productivity across space presents opportunities to maximize biomass production in different geographic regions. For example, combining multiple loci that have frequent benefits and minimal costs together through breeding could produce cultivars that are highly productive across a large geographic region.

To establish how individual loci involved in adaptive divergence are modulated by climatic factors that vary over geographic space, we planted 425 clones from a four-way outbred mapping population (40) at all 10 field sites. At each site, we also planted clones of all four grandparents and F1 hybrid parents of the mapping population. After two full years (spring 2016–spring 2018) of studying these plantings, we were able to address the following major questions: (*i*) How does variation in environmental conditions across geographic space influence adaptive trait variation? (*ii*) How often are QTL involved in adaptive divergence subject to genotype × environment (G×E) interactions? (*iii*) To what extent are there costs associated with adaptive loci when transplanted into various environmental conditions? (*iv*) What are the predicted effects for individual loci and aggregate genotypes on biomass across space?

## Results

### Trait Variation and Fitness across Genotypes and Space.

We observed strong survival-associated local adaptation for the field sites at the northern and southern extremes of our experiment but little differential survival across the middle latitudes. The AP13 (Alamo cultivar, Texas) and WBC3 (wild accession, Texas) southern lowland grandparents both experienced 80.0% mortality at the most northern site in Brookings, SD (*SI Appendix*, Fig. S1). However, there was no mortality of AP13, and only a mean of 4.9% mortality of WBC3, across the other field sites (*SI Appendix*, Fig. S1). Conversely, the northern upland grandparents DAC6 (Dacotah cultivar, North Dakota) and VS16 (Summer cultivar, Nebraska) experienced 83% and 53% mortality across the four southernmost sites (all in Texas) but only 3.8% and 4.1% mortality elsewhere (*SI Appendix*, Fig. S1). Compared with its grandparents, the recombinant four-way mapping population had >7.6 times higher likelihood of survival (Fisher’s exact test *P* < 1 × 10^−16^). Mean mortality of the mapping population genotypes was only 2.1% (95% interquantile range: 0.0 to 12.5%), compared with 14.5% mortality in the grandparents across the 10 field sites.

We also observed strong G×E for biomass (*F*_23,389_ = 29.5, *P* < 1 × 10^−16^) among the grandparental genotypes (Fig. 1*B*). Consistent with local adaptation in the production of biomass, the two southern lowland genotypes, both natives of Texas, achieved maximum biomass in common gardens in Texas, whereas the northern upland genotypes had progressively higher yields going from south to north (*SI Appendix*, Fig. S2). However, the northern uplands never outperformed the southern lowlands in the first two growing seasons (2016 and 2017), as high winter mortality did not occur for the southern lowlands in the north until the 2017/2018 winter (*SI Appendix*, Fig. S1). There was a very significant site effect on biomass production (*F*_9,4455.4_ = 821.1, *P* < 1 × 10^−16^) among the recombinant F_2_ population, where the southern sites generally had lower biomass yield than the northern sites (*SI Appendix*, Fig. S3). Other traits generally followed patterns similar to biomass, with strong site effects of both tiller height and tiller number among the grandparents (height: *F*_9,414.01_ = 82.6, *P* < 1 × 10^−16^; tiller count: *F*_9,375.1_ = 14.7, *P* < 1 × 10^−16^) and F_2_ population (height: *F*_9,4364.6_ = 2507.3, *P* < 1 × 10^−16^; tiller count: *F*_9,3841.4_ = 234.3, *P* < 1 × 10^−16^).

Latitude of planting was an even stronger driver of phenological trait variation than of biomass (*SI Appendix*, Fig. S2). Spring emergence from the rhizome crown (50% “green-up” timing; *F*_9,416.22_ = 602.0, *P* < 1 × 10^−16^) and the timing of flowering (50% of tillers at anthesis, “flowering”; *F*_9,379.02_ = 333.17, *P* < 1 × 10^−16^) were earlier at the southern sites for all four genotypes. There were highly significant differences in flowering (*F*_3,__379.07_ = 473.2, *P* < 1 × 10^−16^) and green-up (*F*_3,416.03_ = 110.5, *P* < 1 × 10^−16^) among the grandparents. In general, the southern lowland grandparents emerged earlier in the season and flowered later in the season. However, the divergence in green-up time between southern lowland and northern upland grandparents became less pronounced at the more northern field sites (Fig. 1).

### QTL of Adaptive Divergence.

**Fig. 2.**
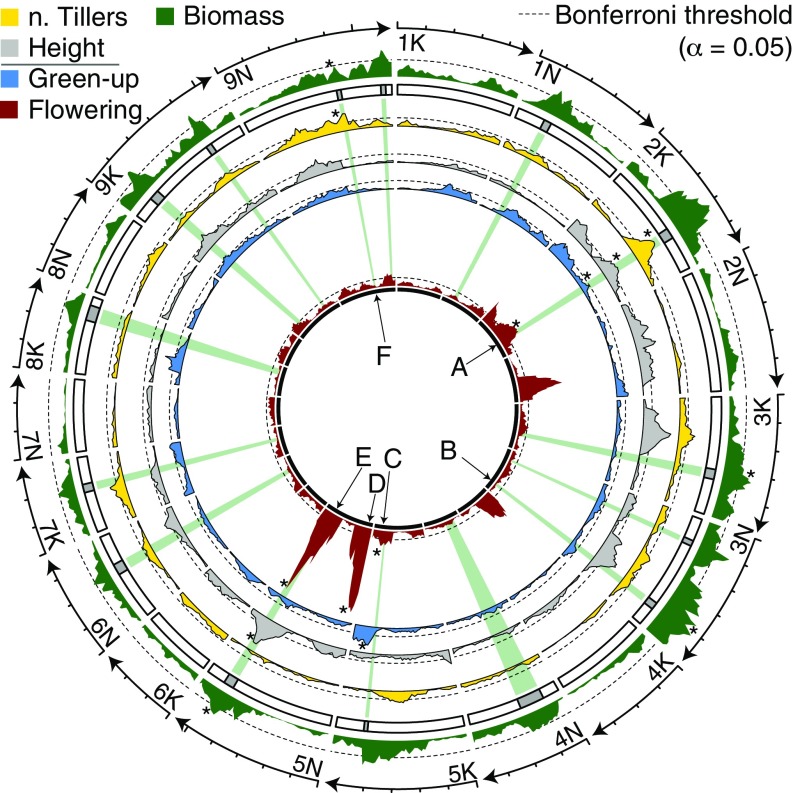
Mapping positions of QTL across five traits. −log_10_ *P* value support for QTL is plotted in each track, where the mapping position (centimorgans) is the *x* axis. Each minor tick on the outer segments indicates 20-cM distance. The primary phenotype, biomass, is presented along the outer track on its own scale. The remaining four phenological and morphologic traits are all on an identical scale. All significant QTL are highlighted from the center as gray rays. Six focal QTL (A–F) are indicated with arrows. The genotypic effects of these QTL are plotted in Fig. 3 following this naming scheme. All significant QTL for each trait are indicated by an asterisk. Plot includes data analyzed across both 2016 and 2017.

We detected multiple significant QTL for all five traits (Fig. 2 and *SI Appendix*, Fig. S4). There were 15 significant QTL for biomass, 19 for flowering time, 14 for spring green-up time, 16 for plant height, and 14 for tiller number for data collected in 2016 and 2017 (*SI Appendix*, Table S1). There were significant G×E effects for 70 (90%) of the QTL (*SI Appendix*, Table S1). These G×E effects include site × year combinations as a component of E. Two flowering time QTL on chromosome 5N dominated the genetic architecture of this trait (*SI Appendix*, Figs. S5 and S6). QTL effects were generally more moderate for the other traits (*SI Appendix*, Figs. S5 and S6).

**Fig. 3.**
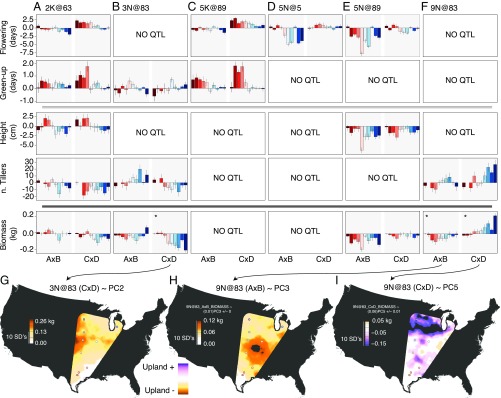
Genotypic effects and climatic correlates of six QTL. (*A*–*F*) The genotypic effect (±SD) for each QTL is presented as the difference between genotypes, when substituting the upland allele for the lowland. These allelic effects are plotted independently for each side of the cross, where effects are displayed as bars arranged from the southernmost (left-red) to northernmost (right-blue) field sites. A×B is the cross between AP13 (lowland) and DAC6 (upland). C×D is the cross between WBC3 (lowland) and VS16 (upland). Positive additive effects indicate that the upland allele increased the trait value, while negative additive effects indicate that the lowland allele increased the size of the trait. QTL× trait combinations with no significant QTL are indicated as such. QTL are named following the chromosome@position convention. (*G*–*I*) The predicted biomass changes of a set of QTL (indicated by asterisks), where climatic principal components were used to model genotypic effects. The empty areas in the prediction surface are either beyond the geographic or climatic scope of the study.

A major outstanding question about the evolution of switchgrass is whether the same loci consistently contribute to the divergence of the upland and lowland ecotypes or whether different loci are responsible for their divergence across the species’ range. The design of the crosses to generate the outbred population (40) allowed us to quantify the differences in effects of AP13 (lowland) vs. DAC6 (upland) alleles and the differences in effects for the WBC3 (lowland) vs. VS16 (upland) alleles simultaneously. These two lowland–upland contrasts let us test the relative prevalence of fixed upland–lowland differences (same directional effect in both contrasts) and those that were private to a single genotype (only significant in one contrast or in opposite directions for the two crosses; *SI Appendix*, Fig. S6). Overall, the direction of significant (≥2 SD from mean) QTL effects was not statistically associated between the two sides of the cross for biomass (binomial probability = 0.51, *P* > 0.1) or tiller count (prob. = 0.54, *P* > 0.1) and only marginally significantly associated among flowering time QTL (prob. = 0.56, *P* = 0.04). However, the two crosses contributed more similarly for green-up and height, where 60 to 62% of the unique QTL effects had effects in the same direction (both *P* values < 0.0001). For example, QTL 5N@89 (height) and QTL 5K@89 (green-up) additive effects were in the same direction for both the A×B and C×D crosses (Fig. 3). Overall, these results suggest that the same loci are not consistently involved in divergence between southern lowland and northern upland ecotypes across their geographic ranges.

### QTL Effects and Magnitude of Trade-offs across Geographic Space.

To evaluate how the additive effects of individual QTL varied across space, we conducted a focused analysis on phenotypes quantified in 2017 (*SI Appendix*, Table S2). We observed large asymmetries in additive effects across different field sites (Fig. 3 *A*–*F*). QTL effects were often found in one geographic region, but not others (*SI Appendix*, Fig. S6). For example, the biomass QTL at 3N@83 and 9N@83 had detectable effects for the northern six field sites but no effects in the four southern field sites (Fig. 3 *B* and *F*). Only seven of the G×E QTL showed a trade-off pattern of allelic effects across geographic space, where the allelic effects changed direction across the range. Further, many G×E QTL did not have linear clinal effects on traits across space. For example, the strongest flowering time QTL (5N@89) had the largest effects on both flowering time and biomass at field sites at midlatitudes, with smaller effects in both the far north and south (Fig. 3*E* and *SI Appendix*, Fig. S6). The partial colocalization of QTL for different traits may be responsible for the correlations in these traits across the outbred mapping population (*SI Appendix*, Fig. S7).

**Fig. 4.**
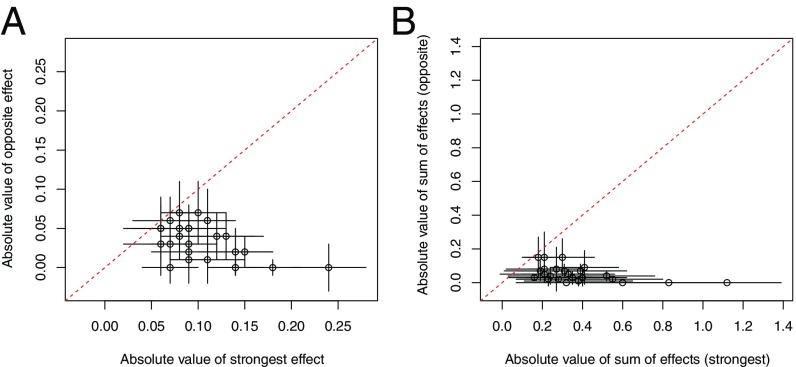
Magnitude of fitness trade-offs for biomass QTL. (*A*) For each biomass QTL, the absolute value of the additive effect of an allele at the best field site (*x* axis) is plotted against the absolute value of additive effect of that same allele at its worst-performing site (*y* axis). For all loci, the allele effects at the worst performing site were either zero or in the opposite direction. (*B*) For each biomass QTL, the sum of additive effects of an allele for all field sites where it is beneficial (*x* axis) is plotted against the sum of additive effects for all field sites where it has effects in the opposite direction on biomass (*y* axis). For both plots, points that are closer the diagonal dashed line represent strong fitness trade-offs, while those closer to zero on the *y* axis have little or no fitness trade-offs. Note that the largest effect QTL show no evidence of fitness trade-offs.

We evaluated the overall extent to which there are trade-offs for individual biomass QTL two different ways. First, we compared the additive effect of each QTL at its best-performing site to its worst-performing site. For all biomass QTL, there was at least one site where there was an additive effect in the opposite direction or no detectable effect. This comparison revealed that QTL ranged from having strong trade-offs to no detectable trade-offs (Fig. 4*A*). We then compared the sum of additive effects at all sites where the QTL had a beneficial effect to the sum of all of the additive effects across sites with allelic effects in the opposite direction. This comparison, which considers all 10 sites jointly, suggests that trade-offs do occur but are generally rare (Fig. 4*B*). In addition to the evaluation of individual QTL, we calculated pairwise genetic correlations for biomass among field sites for the four-way outbred population. Our expectation was that if there were strong fitness trade-offs for loci controlling biomass that there should be a negative genetic correlation for biomass among some of the field sites. Instead, we observed only strong positive correlations for all pairwise comparisons (*SI Appendix*, Fig. S8).

### Modeling QTL Effects across Geographic and Climatic Space.

One of the key advantages of conducting our experiment at 10 field sites was that we captured the climate variability relevant to >80% of the latitudinal range of switchgrass in the United States (Fig. 1). This design therefore allowed us to model the allelic effects of QTL across much of the spatial and climatic range of switchgrass. To predict additive allele effects across space, we first modeled the additive effects for each QTL as a response to the principal components of a set of climate variables (*SI Appendix*, Fig. S9 and Tables S3–S5). We then used these predictive models to create an interpolated raster surface of the predicted additive effects for each QTL across a triangular region defined by a maximum extent 200 km beyond any of the 10 field sites.

Since most biomass QTL had effects that varied greatly across space, it is not surprising that the interpolated effect of these QTL varied significantly across climatic gradients (Fig. 3 *G*–*I* and *SI Appendix*, Fig. S10 and Tables S6 and S7). For example, the WBC3 and AP13 (both lowland) alleles for QTL 3N@83 and 9N@83, respectively, had large biomass-increasing effects only in the north and central regions. Conversely, the upland VS16 allele of 9N@83 improved biomass by >200 g per plant in the far north but rarely by more than 50 g elsewhere. It is important to note that the geographic distribution of effects for the two lowland alleles at 9N@83 QTL were opposite, where the AP13 allele improved biomass (especially in the middle of the range) while the WBC3 allele reduced fitness dramatically in the north (Fig. 3*F*). Since it is clear that not all QTL act consistently between upland and lowland genotypes, our results illustrate the importance of testing multiple alleles per ecotype.

**Fig. 5.**
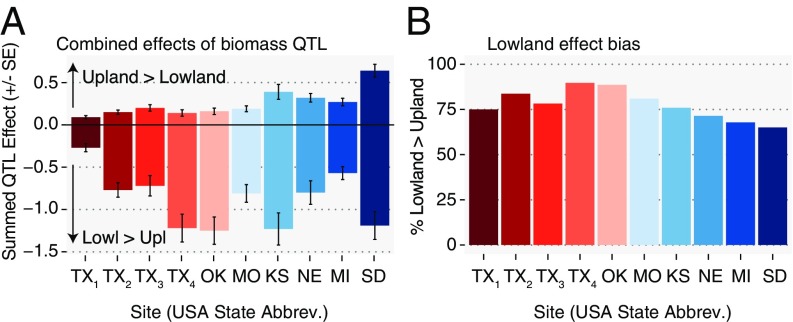
How upland and lowland alleles contribute to the optimal genotype at each field site. (*A*) The bar plots above zero correspond to the summed effects of alleles across loci, where the upland allele made plants larger. Bar plots below zero correspond to the summed effects of alleles across loci, where the lowland allele made plants larger. (*B*) The percentage of the overall biomass increase caused by lowland alleles for the optimal genotype at each field site. To calculate values, the genotypic effects of all significant QTL (effect >2 SE from zero) at each site were extracted. The predicted effects and SEs of these QTL were then multiplied by the sign of the effect at each site and summed for each site.

A major goal of switchgrass breeding programs is to develop regionally adapted cultivars that maximize biomass production for different regions. To further this aim, we assembled hypothetical genotypes that were aggregate combinations of detected alleles that maximized biomass at each of the 10 field site locations for data from 2017. From this analysis, we found that southern lowland alleles always contributed more additive effects than upland alleles across all sites (Fig. 5). However, the total (Fig. 5*A*) and relative (Fig. 5*B*) contributions of northern upland alleles were greater in the more northern sites. These are likely good estimates of combined allelic effects, as fivefold cross-validation of our models had considerable prediction accuracy when averaged across all 10 tested sites (rms error = 0.591; percent bias = −0.3%; *r* = 0.601; *SI Appendix*, Fig. S11). The estimated combined effects of QTL, assuming only additive effects, were largest for the Brookings site (a potential increase of 1.7 kg per plant per y).

## Discussion

With its unprecedented scale, our study in switchgrass provides a clearer picture of how individual loci contribute to adaptive trait variation across geographic space. The vast majority of QTL had significant G×E effects, indicating that environmental context is critically important in interpreting quantitative genetic results. We found that there were trade-offs for some biomass QTL, but those trade-offs were typically weak or only occurred at a small minority of field site across the 2 y of our study. We leveraged climate modeling to predict the effect of individual loci across geographic space as well as the combined effects of those loci across our 10 field sites. Overall, these results clarify how adaptive trait variation across large-scale environmental gradients is controlled by a combination of genes and the environment. We discuss these results below in the context of previous studies of the genetics of local adaptation in switchgrass and other organisms.

### Patterns and Genetics of Local Adaptation.

Consistent with previous studies of switchgrass (26, 29, 38, 39), there was a strong pattern of local adaptation between northern upland and southern lowland ecotypes in terms of survival. The high mortality of the northern upland grandparents across the southern sites is likely the result of stress imposed by high temperatures and other environmental factors, such as pathogen load (41). In contrast, the high level of mortality of the southern lowland grandparents at the Brookings site was due to winter kill. The vast majority of winter kill of AP13 (100%) and WBC3 (83%) at Brookings occurred in the 2017/2018 winter, which was significantly colder than the previous two winters. Future data collection from our experiment will clarify how that harsh winter translates to impacts on biomass production across the northern sites. Similar variability in the impact of winter on fitness was found in a multiyear study of local adaptation of *Arabidopsis thaliana* between field sites in Italy and Sweden (17). In that study, selection in the hotter, drier site in Italy was consistently significant across years, while local selection against foreign transplants due to cold temperatures in Sweden was only detectable in three out of five field seasons. Taken together, these studies suggest that selection in the north, caused by winter damage/kill, may generally be a more variable source of stress than selection at southern sites in both Europe and eastern North America.

Field QTL studies of local adaptation frequently find strong effects of individual loci at one field site but not in the alternative site (4, 5, 19). Similar results have been found for field genome-wide association studies. For example, Fournier-Level et al. (20) found that out of the 797 top SNPs associated with local fitness, only 12 were detected at more than one site. If strong fitness trade-offs were common at individual loci, we would expect more loci to have detectable effects across field sites. While evaluation of the top SNPs did not support widespread strong trade-offs, Fournier-Level et al. (20) did potentially find evidence for trade-offs with a weak negative correlation of SNPs influencing survival for comparisons among pairs of field sites.

Our study found that a few QTL had strong fitness trade-offs, if only the best and worst sites were compared (Fig. 4*A*). However, trade-offs were much reduced when positive and negative effects are summed across sites (Fig. 4*B*). This result is caused by trade-offs either being weak, found at only a few field sites, or undetectable. Our finding that there were only strong positive genetic correlations of biomass across field sites (*SI Appendix*, Fig. S8) also suggests that the magnitude of the collective fitness trade-offs of loci across the genome is not nearly as great as their benefit.

The finding that trade-offs are generally weak implies that gene flow is restricted between upland and lowland switchgrass ecotypes. This is because only loci that cause trade-offs in fitness effects across habitats are predicted to be restricted to alternative habitats if there is appreciable gene flow between locally adapted populations (6–9). In contrast, alleles with little or no fitness costs are expected to be spread across habitats by gene flow (4, 9, 23, 42). Previous studies of the population genetics of switchgrass have found moderate levels of population structure between switchgrass ecotypes (*F*_*ST*_ = 0.048 to 0.096; refs. 25, 43, and 44), which could be enough to allow for the evolution of local adaptation through loci with little or no fitness trade-offs. Restrictions on gene flow among switchgrass populations could also explain why some lowland alleles have greater effects on fitness in the north than the south (e.g., the 3N@83 QTL; Fig. 3*G*). Such alleles may represent species-wide selective sweeps that are in their early stages. Similarly, Latta (19) discovered that a major fitness QTL in *Avena barabata* appears to be in the early stages of a selective sweep.

Despite population structure between ecotypes, we found little evidence that the same set of loci was consistently responsible for divergence between upland and lowland switchgrass ecotypes. In our study, only a small number of QTL had similar allele effects for the A×B and C×D sides of the cross that formed the outbred mapping population. This suggests that while upland and lowland ecotypes are genetically and morphologically distinct, different loci contribute to adaptive ecotype divergence across the range of the species. In contrast, studies that only use biparental crosses to study local adaptation can result in a biased interpretation of which loci are involved in broader patterns of adaptive divergence (16, 22). We urge future researchers to use more individuals in their crosses or make replicated crosses between different populations (45) to test whether individual loci have consistent effects on locally adaptive divergence across the range of species.

### Strategies for Breeding Regionally Adapted Cultivars.

Our results suggest that climate modeling of additive effects of QTL across space offers an excellent opportunity to exploit locally adapted traits for developing regionally adapted cultivars. Because trade-offs were generally weak, rare, or nonexistent for biomass QTL across space, there is tremendous opportunity to breed high-yielding lines that perform well across large geographic regions. The greatest gains for biomass seem to be in the far north, where multiple QTL with effects in the same direction as the parental divergence could be combined with multiple QTL that have effects in the opposite direction of the parental divergence (i.e., northern upland allele increasing size and vigor). An outstanding question is why these QTL in the north have effects on biomass in the opposite direction of the parental divergence. One possibility is that these QTL are involved in cold tolerance. The ultimate cause of that tolerance will need to be worked out with more detailed studies, as perennial plants can be damaged due to mistiming of fall senescence, tolerance to freezing of overwintering rhizomes, or tolerance to chilling after emergence of aboveground tillers in the spring (34).

Despite the finding that multiple loci could be combined to greatly increase yield, it is unclear whether a “jack-of-all-trades” cultivar that maximizes biomass yield in all locations could be developed. The patterns of additive effects clearly differ for the maximization of yield in different geographic regions (Fig. 3). Further, some of the loci might have much stronger trade-off in years with different weather conditions, as was the case for *A. thaliana* transplants between Italy and Sweden (17, 22, 23). This is an important caveat, as we have only so far quantified biomass in years (2016 and 2017) that followed relatively mild winters in the north. Continued analyses of these gardens over time will provide more clarity into whether stronger trade-offs do emerge in years that follow harsher winters.

## Conclusions

Overall, our results suggest that loci with highly variable effects across climatic conditions drive local adaptation in switchgrass. This variation, once quantified, could be exploited to breed cultivars with adaptations to a broad range of environmental conditions in switchgrass and other crop species. Our results suggest a need for an expansion of research into the genetics of local adaptation beyond two-site reciprocal transplant experiments, especially to situations with less restricted gene flow, where strong trade-off loci are more likely to dominate the genetic architecture.

## Methods

### Experimental Design and Phenotyping.

The details of creation of the genetic mapping population are described in Milano et al. (40). Briefly, the genetic mapping population was produced by initial crosses between AP13 × DAC6 (A×B) and WBC3 × VS16 (C×D). The F1 hybrids of each of those crosses were then intercrossed reciprocally to produce the four-way outbred mapping population. The four-way population, grandparents, and F1 parents were propagated clonally in 3.8-L pots at the Brackenridge Field Laboratory, Austin, TX in 2014–2015.

Plants were transported to each of the 10 field sites by truck and planted at each site in May–July of 2015. Each field was covered with one layer of weed cloth (DeWitt). Holes were cut into the weed cloth for planting of the experimental plants. Plants were randomized haphazardly into a honeycomb design, where each plant had four nearest neighbors, all located at 1.56 m away from each other. To prevent edge effects, a row of plants derived from the lowland Alamo cultivar were planted at every edge position of the plot. Plants were hand-watered following transplantation as needed through the summer of 2015. Plants were not measured until the spring of 2016 to allow them to become established through one winter first.

The five phenotypes for this study were assessed as follows. Green-up time was scored as the Julian date at which point a plant had sprouted new tillers from 50% of the area of the crown from the previous season. Flowering time was scored as the point when 50% of the tillers of the plant had panicles undergoing anthesis. The number of green tillers were counted within a few weeks after the 50% flowering date. Height was measured from the base of the plant to the uppermost point of the canopy. At the end of each season, plants were tied upright as a bunch and harvested with a sickle bar mower. Wet biomass was quantified in the field. A subsample of each plant was also weighed and then dried at 55 °C until completely dry and weighed again. Percent water content for each subsample was then used to calculate the dry biomass of each plant.

### Genotyping and Map Construction.

In brief, Illumina libraries from each of the four grandparents were aligned to the *P. virgatum* V4 reference genome via bwa mem (46) and used for single-nucleotide polymorphism (SNP) calling (mpileup2snp-Varscan2; ref. 47). SNP positions were used to create 64-bp nonoverlapping windows (64-mers). Unlike biallelic markers (e.g., SNPs), this kmer-based approach captured multiple variants and allowed us to uniquely distinguish each grandparent when genotyping the progeny. Of 10,734,933 possible kmers, 4,122,301 contained enough read coverage to generate kmers from three of four grandparents, which were then typed in the 431 progeny. After removing nonunique kmers (e.g., those shared among upland grandparents) and those with >60% missing data, 263,776 markers were retained.

The resulting genotype matrix was binned via sliding windows across the physical V4 Switchgrass genome positions, where the majority genotype was retained for each progeny within each 50-marker window. Linkage groups were formed from this culled sliding window genotype matrix from pairwise recombination fractions, where all pairwise recombination fractions among markers within a linkage group must be <0.25. Markers were ordered within linkage groups with the traveling salesman problem solver Concorde (48), which finds the path among markers that limits total recombination fractions per linkage group (49). This approach is more accurate and faster than other linkage mapping protocols for NGS data (49). Map polishing and fine-scale reordering was accomplished in R/qtl via the ripple algorithm (50).

### Multienvironment/Year QTL Mapping.

We studied 425 recombinant progeny grown at 10 locations for two consecutive years (2016 and 2017). Before QTL mapping the average values of the phenotypes were calculated for genotypes that had multiple observations of phenotypes at a given site for each year. This resulted in only one single value for each genotype and for each phenotype at a given site. Outlier phenotypic observations were subsequently culled based on the linear model: phenotype = line + site + line * site. Points with residuals with *t*-distribution Bonferroni-corrected *P* values < 0.05 were discarded (51). QTL mapping was conducted with Genstat to identify QTL as well as QTL × E interactions (52). Data of the cross-pollinated population from across the 20 site-by-year combinations were fit into a multienvironment trials model following the methods of Malosetti et al. (53). Briefly, this model consisted of the population mean (*µ*), a fixed effect of environment (*E*), a random genotype effect (*g*), and a random effect of genotype-by-environment (*g x E*) as shown in Eq. **1**:

trait=μ+E+g+g x E+e. [1]

An unstructured model was selected to represent the variance–covariance components and was later used to specify the data structure in a genome-wide QTL scan using simple interval mapping, which evaluates each marker individually for significance (54). Then, the QTL identified from simple interval mapping were specified as cofactors in composite interval mapping (CIM), and CIM was run three times consecutively to confirm stability of the fitted statistics profile.

In our study, the minimum separation distance for selected QTL was set to 30 cM and the minimum cofactor proximity was set to 50 cM based on current linkage map information. Genome-wide QTL significance was assessed at *α* = 0.05, using a Bonferroni correction based on the number of effectively independent tests (55). The fitted model from Eq. **1** contained all significant QTL and QTL × E terms, with both parent additive effects (the first and second parent) and dominance effects, as shown in Eq. **2**:

trait=μ+E+∑QTL+∑(QTL x E)+e, [2]

where *µ* is the population mean; *E* represents the environment effect; $\sum QTL=\sum \left(\alpha^{a}+\alpha^{a2}+\alpha^{d}\right)$, which represents the total effect of each QTL, including the additive effect from the first parent $\left(\alpha^{a}\right)$, the second parent $\left(\alpha^{a2}\right)$, and the dominance effect $\left(\alpha^{d}\right)$; ∑(QTL x E) is total QTL × environment interactions; and *e* represents the error term that was modeled by the unstructured variance–covariance matrix. A backward selection procedure was used to retain significant fixed terms (*P* < 0.05). The above procedure was implemented in GenStat v.19 (52).

### Heritability Estimates.

We estimated narrow-sense heritability *h*^2^ as $\sigma_{A}^{2}/\sigma_{P}^{2}$, where $\sigma_{A}^{2}$ is the additive variance and $\sigma_{P}^{2}$ is the total phenotypic variance (*SI Appendix*, Fig. S2). We estimated $\sigma_{A}^{2}$ for each trait using the additive relationship matrix from kinship based on marker genotypes from resequencing. The process was implemented using the Sommer package in R (56). Sommer provides reliable multivariate mixed models for different genetic and nongenetic analysis in diploid and polyploidy organisms. The core function mmer solves the mixed model equations proposed by Henderson (57): y=Xβ+Zμ+ε, where *y* is a vector of trait phenotypes, *β* is a vector of fixed effects, μ is a vector of random effects, and *ε* are the residuals. The random effect μ is assumed to be normally distributed with a mean of zero. X and Z are incidence matrices for fixed and random effects, respectively. In the case of our four-way population, the model takes Z and the kinship matrices (i.e., additive relationship matrix estimated from the kinship matrix) for random effects and estimates the variance components for each trait. Genetic correlations were estimated through an identical mixed-effects model as heritability, except that the response variable (*y*) was a bivariate set of BIOMASS from two unique site-by-year combinations. This model was fit iteratively for all pairwise combinations of sites (*n* = 10) and years (*n* = 2). Genetic covariance between response variables were extracted from these models and converted to correlations via the base R function cov2cor.

### Climate Analysis.

To understand the climatic drivers of G×E, we analyzed daily weather data from weather stations at each site and those monitored by the US National Oceanographic and Atmospheric Administration (NOAA; ref. 58). To develop climatic envelopes that accurately represent the weather perceived by plants at each site, we needed to control for the relative timing of growing season at each site. For example, February–March represents the early season for plants in South Texas. However, plants in South Dakota would be dormant during this period of time (*SI Appendix*, Table S3). Therefore, we first inferred the date of first green-up and last flowering for the four-way mapping population at each site. To predict these phenological dates across the 651 NOAA weather stations within our study area, we conducted a scaled and centered principal component analysis (PCA) among all daily mean temperatures (minimum and maximum daily) and mean precipitation variables (*SI Appendix*, Figs. S12 and S13). The first 11 PCA axes cumulatively explained >90% of the total variation (*SI Appendix*, Fig. S13). We then chose the best (lowest Bayesian information criterion; *SI Appendix*, Fig. S14) linear combinations of up to four of these variables that maximized the variance explained through the regsubsets function in the leaps R package (59). These models were highly predictive: *r*^2^ values were 0.996 and 0.900 for green-up and flowering, respectively (*SI Appendix*, Fig. S15). We predicted growing season based on these statistics for 651 NOAA weather stations that were within a minimum convex polygon (hull) that buffered the 10 gardens by 200 km. We interpolated monthly weather statistics across the landscape via inverse distance weighting (*SI Appendix*, Fig. S16; ref. 60).

To define growing season-informed estimates of climatic variables, we subsetted the growing season into three equal-length, 5-d overlapping intervals at each of the 10 sites, running from 14 d before the first observed green-up to 14 d after the last the last plant flowered in the mapping population. We calculated seven statistics for each interval: 95th quantile maximum temperature; fifth quantile minimum temperature; average daily precipitation; and the hottest, coldest, driest and wettest 14-d periods (*SI Appendix*, Table S5). The resultant 21 variables were summarized via principal component analysis in R prcomp, after conducting k = 5 nearest neighbor imputations of missing data via knn.impute in the R package bnstruct (61, 62). The first five eigenvectors, which each explained >5% of the total variation (all five combined to explain >75% of total variation) were extracted (*SI Appendix*, Fig. S9).

### QTL–Climate Modeling.

To evaluate how climatic factors modify QTL effects we conducted a meta-analysis using a meta-regression framework. This analysis was conducted only with data collected in 2017. For each QTL and cross direction, we ran a linear metaregression model selection pipeline, seeking to retain the single most significant PCA axis predictors. Metaregression was implemented in the R package metafor as rma.uni (63). This finds the PCA axis that explains most of the variation in QTL effects sizes within a meta-regression model, where the SE of the QTL estimate (independent variable) from each location was accounted for in the linear model.

We were conservative in determining QTL–climate associations—only Benjamini–Hochberg false discovery rate-corrected *P* values < 0.05/5 (5 represents the number PCA axes and thus the number of independent models fit) were determined as significant (*SI Appendix*, Tables S6 and S7). The resulting models were used to predict QTL effects across the 651 NOAA weather stations and an interpolated raster was built using the same method that was employed to predict growing season length. To be conservative, we did not report extrapolated predictions beyond the geographic or phenotypic distributions observed at the 10 sites. Instead, we only report the geographic distribution of effects that were within 2 SEs of the distribution of observed QTL effects at the 10 sites. As a result, we did not estimate effects for the entirety of the triangular geographic region for all loci (Fig. 3 *H* and *I* and *SI Appendix*, Figs. S9 and S10).

## Supplementary Material

Supplementary File
