# Supplementary material for: QTL × environment interactions underlie adaptive divergence in switchgrass across a large latitudinal gradient
Source: Proc Natl Acad Sci U S A. 2019 Jun 10;116(26):12933–41. doi: 10.1073/pnas.1821543116 (PMC6600931; doi:10.1073/pnas.1821543116)
Supplement: Supplementary File [file pnas.1821543116.sapp.pdf]

PNAS
www.pnas.org

Supplementary Information for

QTL x environment interactions underlie adaptive divergence in switchgrass across a large latitudinal gradient

David B. Lowry, John T. Lovell, Li Zhang, Jason Bonnette, Philip A. Fay, Robert B. Mitchell, John Lloyd-Reilley, Arvid R. Boe, Yanqi Wu, Francis M. Rouquette Jr., Richard L. Wynia, Xiaoyu Weng, Kathrine D. Behrman, Adam Healey, Kerrie Barry, Anna Lipzen, Diane Bauer, Aditi Sharma, Jerry Jenkins, Jeremy Schmutz, Felix B. Fritschi, Thomas E. Juenger

Email: tjuenger@mail.utexas.edu

This PDF file includes:

Figs. S1 to S16
Tables S1 to S7

www.pnas.org/cgi/doi/10.1073/pnas.1821543116

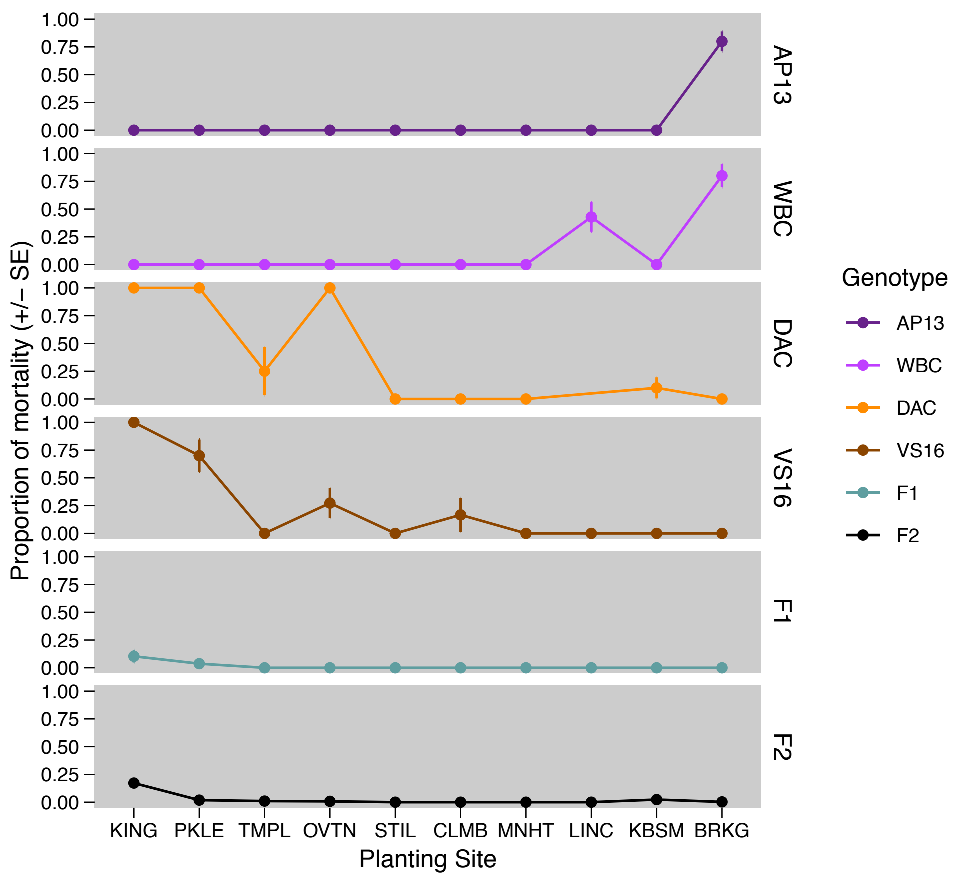

Fig. S1. The proportion of mortality across the 10 field sites, as of the spring of 2018, for each of the four grandparental genotypes, the F1 hybrids, and the outbred hybrid mapping population. Proportion +/- standard error are presented. Sites are ordered as in Figure 1, by increasing latitude.

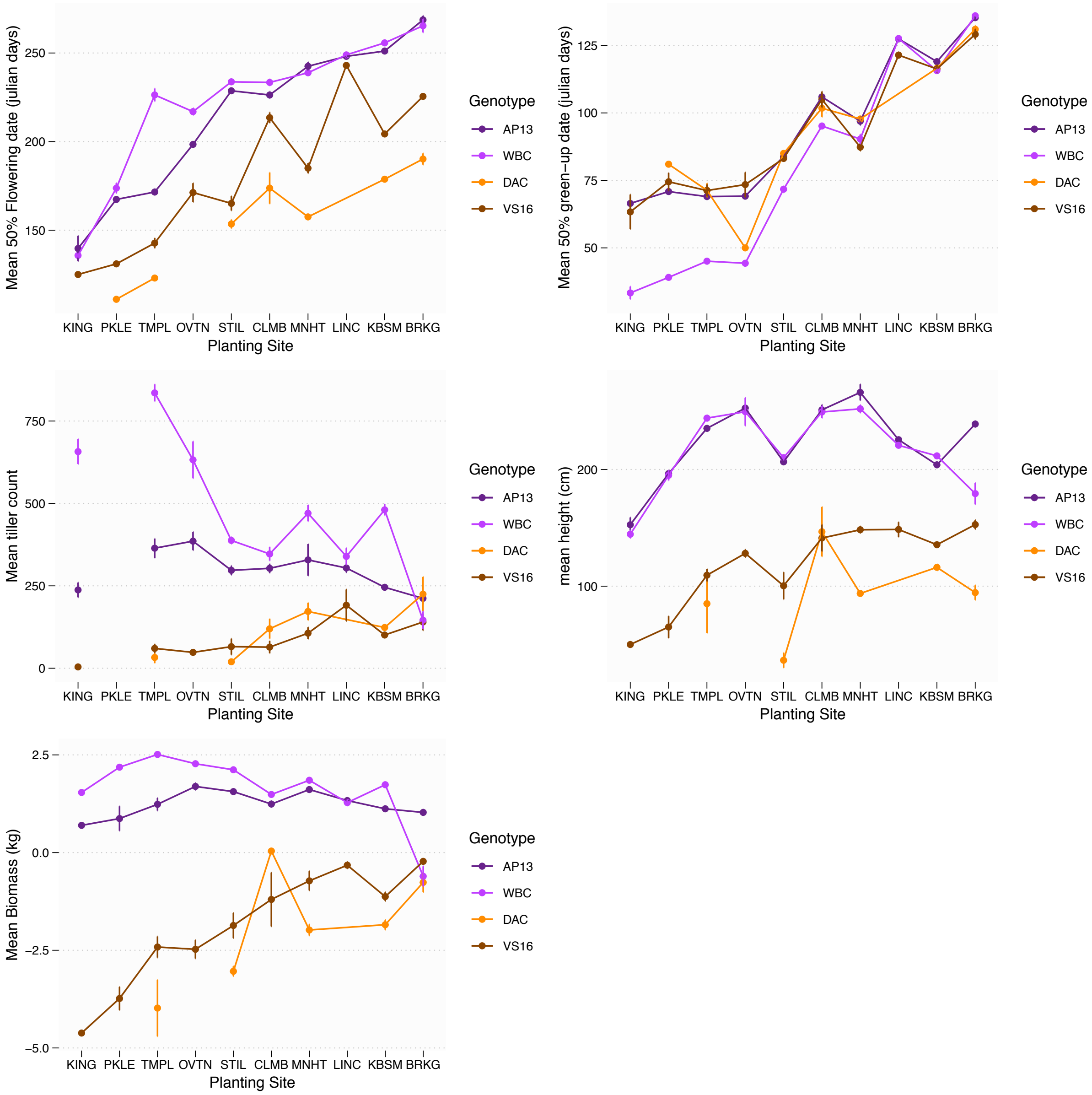

Fig. S2. The distribution of five traits across the 10 sites for each of the four grandparental genotypes. Sites are ordered as in Fig. 1, by increasing latitude. Data are presented for the 2017 harvest only.

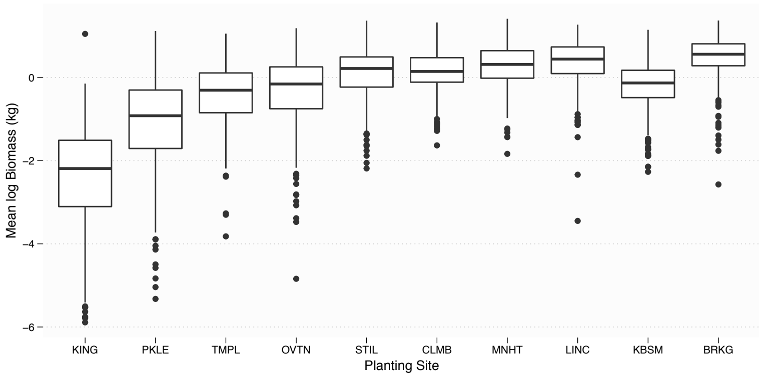

Fig. S3. The distribution of biomass (natural log transformed) in kilograms across the outbred mapping population at each site. Boxes represent the interquartile range. Sites are ordered as in Figure 1, by increasing latitude. Data are presented for the 2017 harvest only.

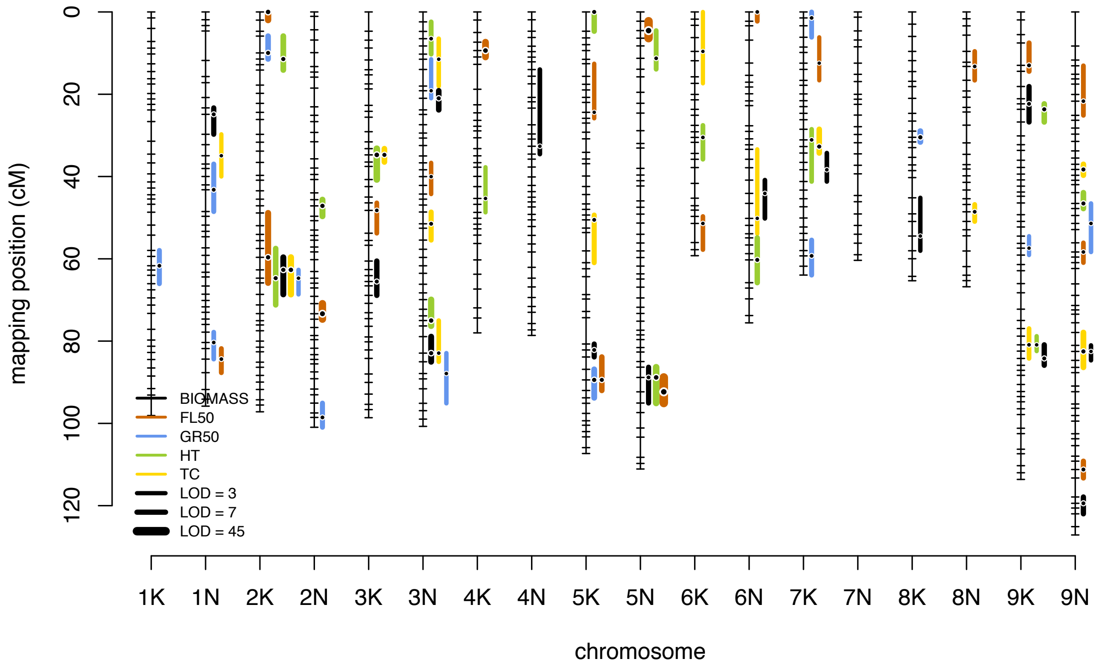

Fig. S4. The location of QTL for all five traits across the switchgrass genome. QTL peaks are presented as black/white points on the assembled linkage map. Vertical lines around these peaks represent the QTL confidence interval, defined as the regions where the profile declines by ≤ 1 LOD. QTL estimates and confidence intervals are from the across-years (2016 and 2017) QTL model.

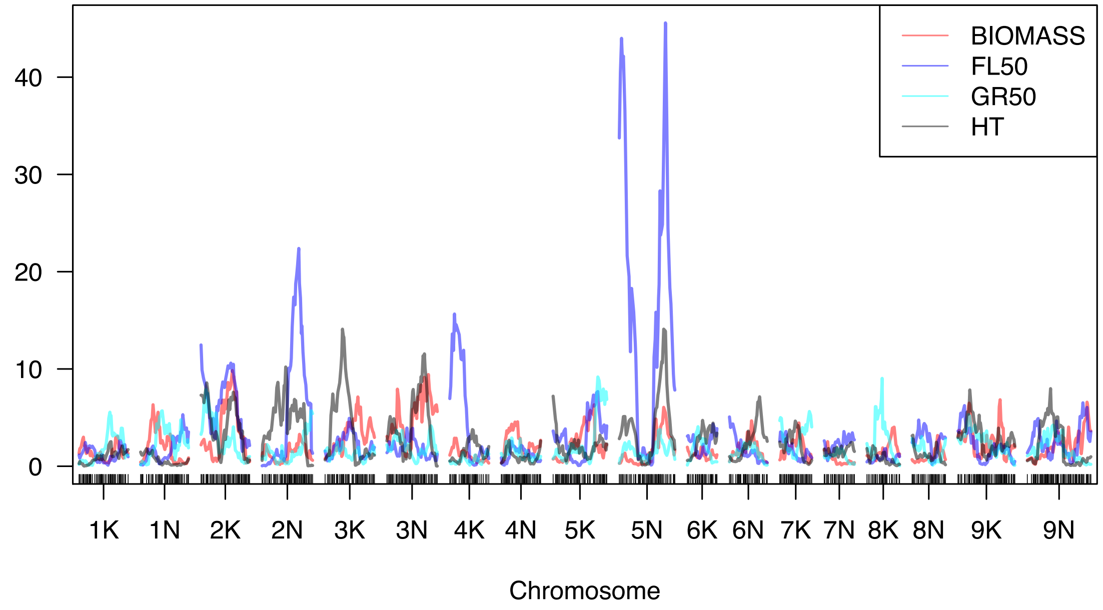

Fig. S5. Genstat-derived -$\log_{10}$ *P*-value profiles for the four of the focal traits along the 18 *Panicum virgatum* linkage groups. QTL estimates and confidence intervals are from the across-years (2016 and 2017) QTL model.

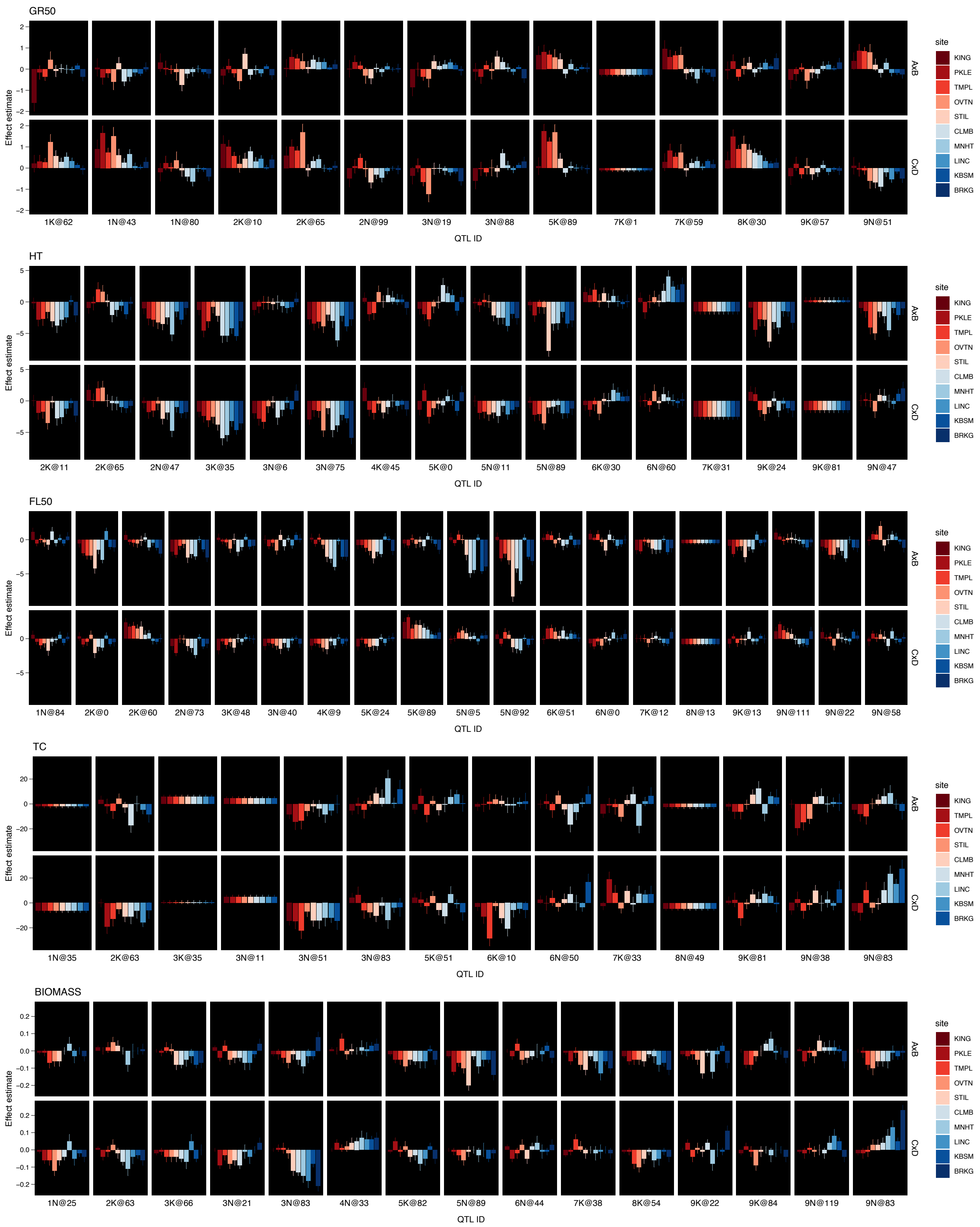

Fig. S6. The QTL effect distribution across the five traits. Sites are colored following Fig. 1. Estimates +/- SE are presented for each side of the cross and each QTL. See Fig. 3 for more details about interpretation of the additive effects displayed in the figure. Effects from the full across-year model were extract, but only effects in 2017 are presented.

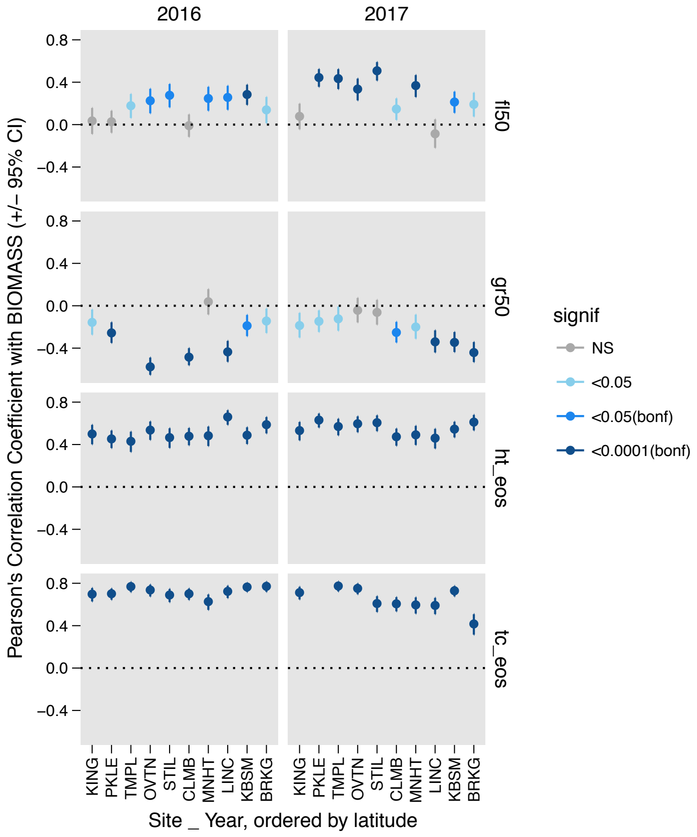

Fig. S7. Phenotypic correlations between biomass and each of the four other traits in the 4-way recombinant mapping population. R values +/- 95% confidence intervals are plotted. Significance is determined by P-values, where those that are marginally significant (uncorrected P-value <= 0.05) are in light blue, while dark blue estimates are significant after Bonferroni multiple-test corrections. Data are from the 2017 harvest.

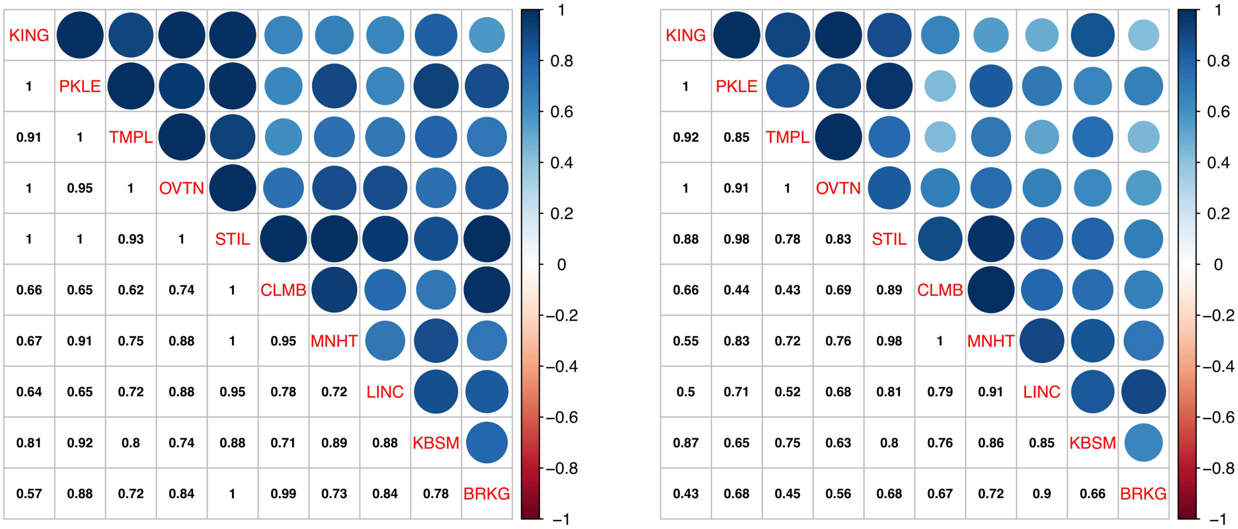

Fig. S8. Pairwise genetic correlations for biomass across field sites for the 4-way recombinant mapping population. All correlations were positive across for both the 2016 (left) and 2017 (right) field seasons.

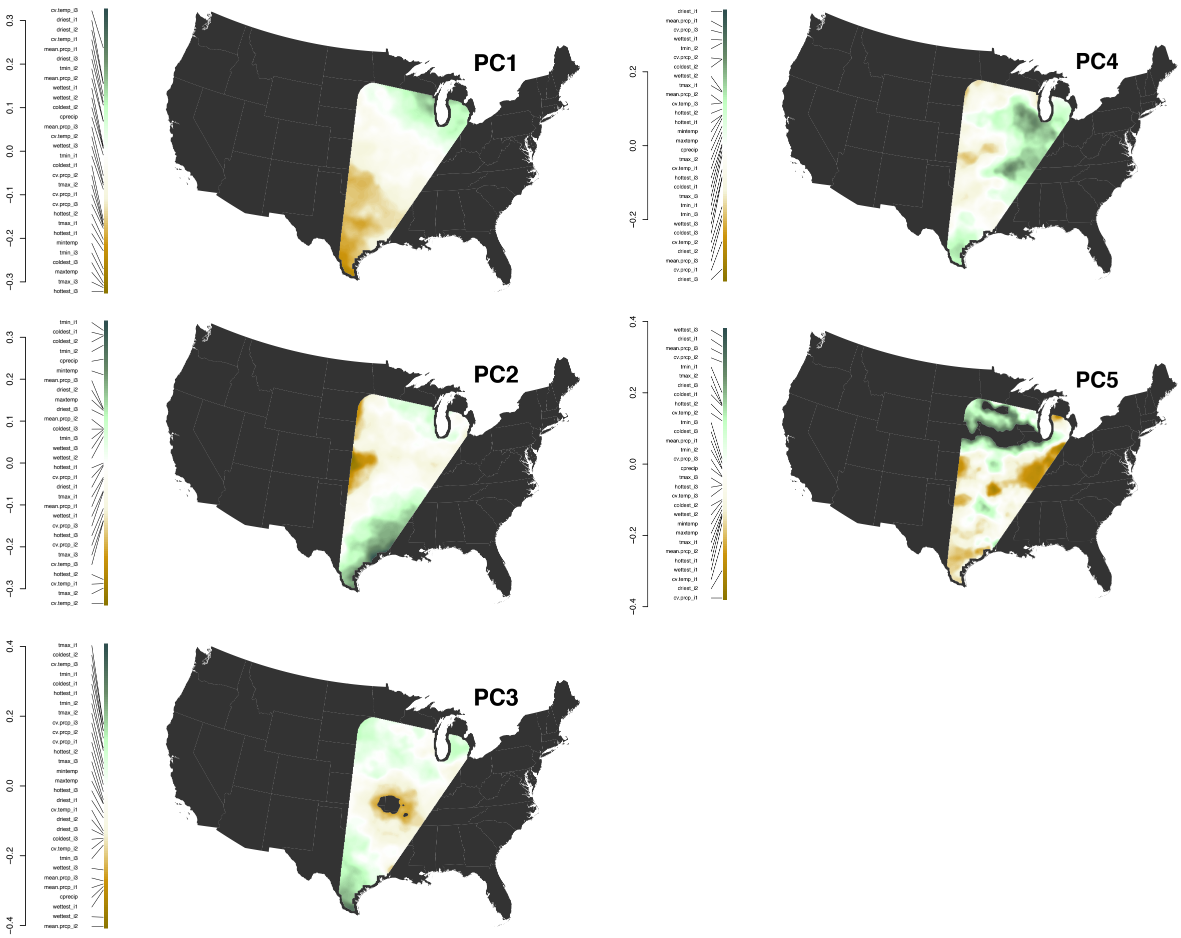

Fig. S9. Spatial distribution of the top five climatic principal component (PC) axes. The significance of each of these as predictors for QTL effects are presented in Table S6. Values outside the range of values observed at the 10 sites (+/- 20%) are masked.

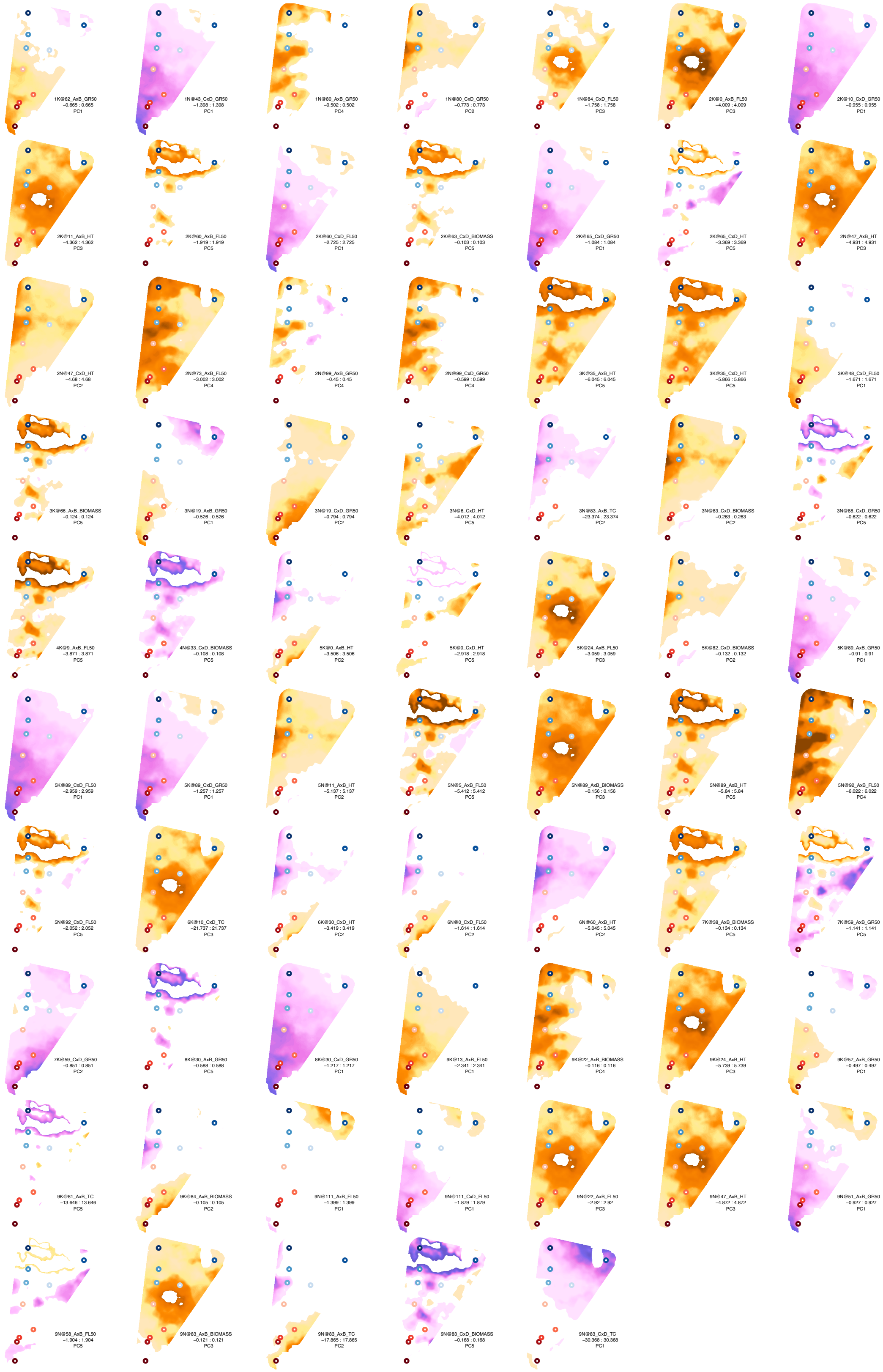

Fig. S10. Predicted QTL effects for all 68 QTL with significant effect-climate relationships, as in Fig. 3.

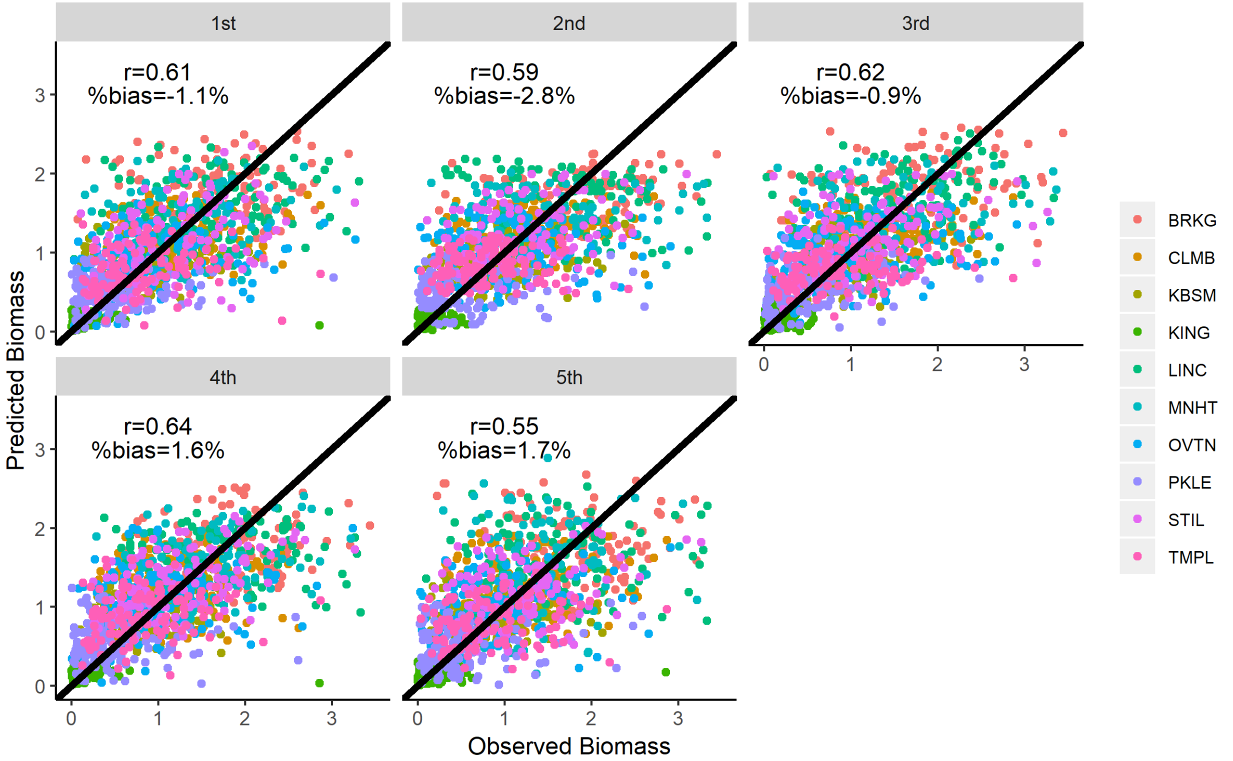

Fig. S11. The quality of the predictions from the full Genotype x Environment (G x E) model was evaluated using a fivefold cross-validation technique. Specifically, we split the individuals, along with their genotypic and phenotypic information, into a training set (80% of the population) and validation set (the remaining 20% of the population). The training set was used to construct the G x E model and the validation set was used to evaluate the model as an independent dataset. The G x E model was extracted from Genstat runs and reconstructed in R to predict biomass for the validation genotypes based on the additive and dominance effects. Root mean square error (rmse), percentage of bias (bias%), and the prediction accuracy (*r*) between the model predictions and field observations were used as statistical measures for model performance. The fivefold cross validation for biomass had an average rmse of 0.591, %bias of -0.3%, and prediction accuracy of 0.601.

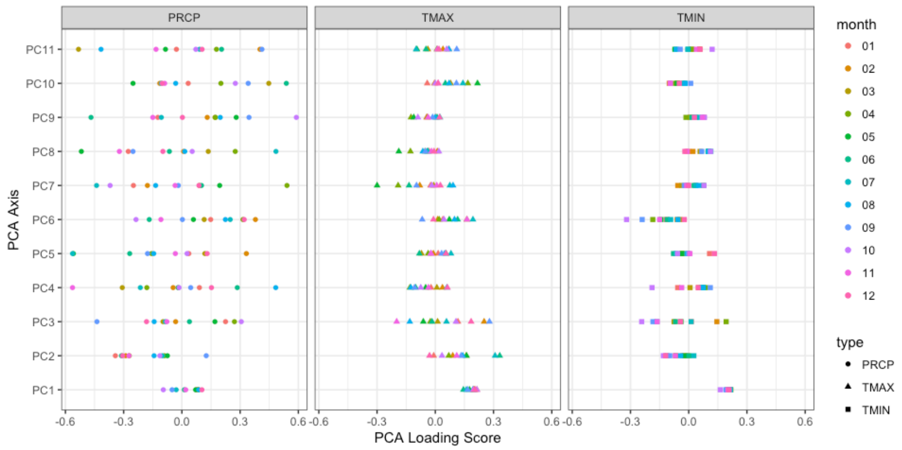

Fig. S12. PCA loadings of the monthly weather data for prediction of the beginning and end of growing season. The month of data collection is indicated by its two-digit numeric code following the variable tested. For example, 95th quantile of maximum temperature in September is specified as TMAX_09.

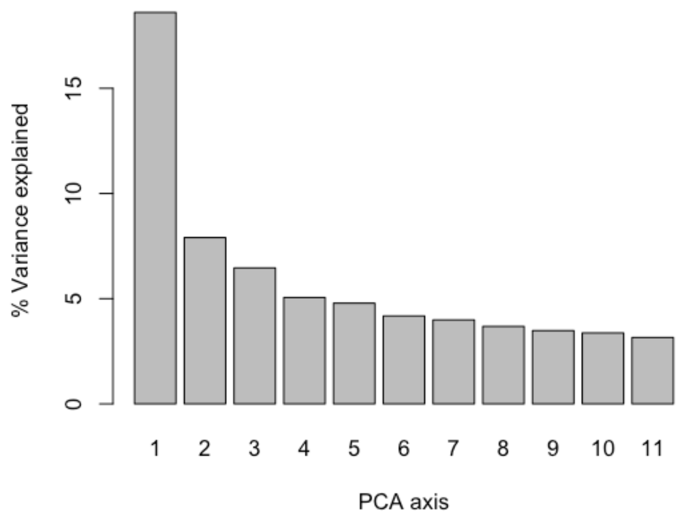

Fig. S13. Percent variance explained of PCA loadings of the monthly weather data for prediction of the beginning and end of growing season.

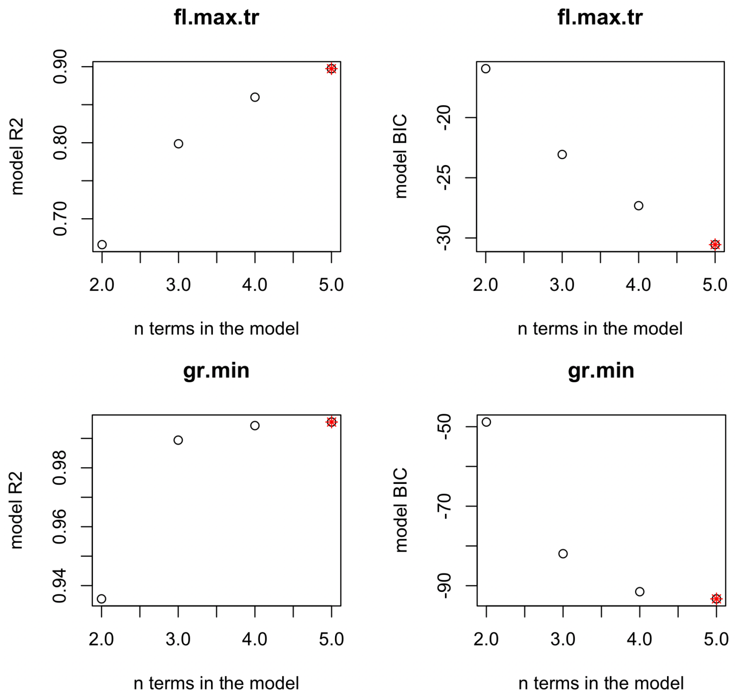

Fig. S14. Selection of the best model for growing season. We employed an iterative model-selection protocol, where we chose the best model (lowest BIC) among up to 4 PCA eigenvectors calculated from the monthly weather variables (n terms also include the intercept).

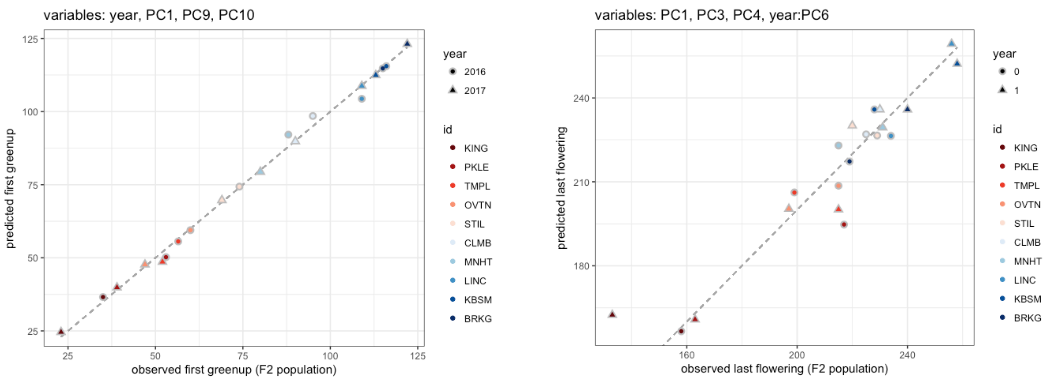

Fig. S15. Prediction of growing season start (left) and end (right). Observed (x) and predicted (y) data are presented for each year (2016 = “0” are circles, 2017 = “1” are triangles. Site colors match those in Figure 1. Note the extremely strong prediction accuracy for greenup time and the slighly lower accuracy for time of last flowering. This indicates that greenup is driven by a linear combination of climatic variables that are well captured by our principal components. However, higher-order interactions, or climate variables that were not present in our data also likely predict the last flowering time.

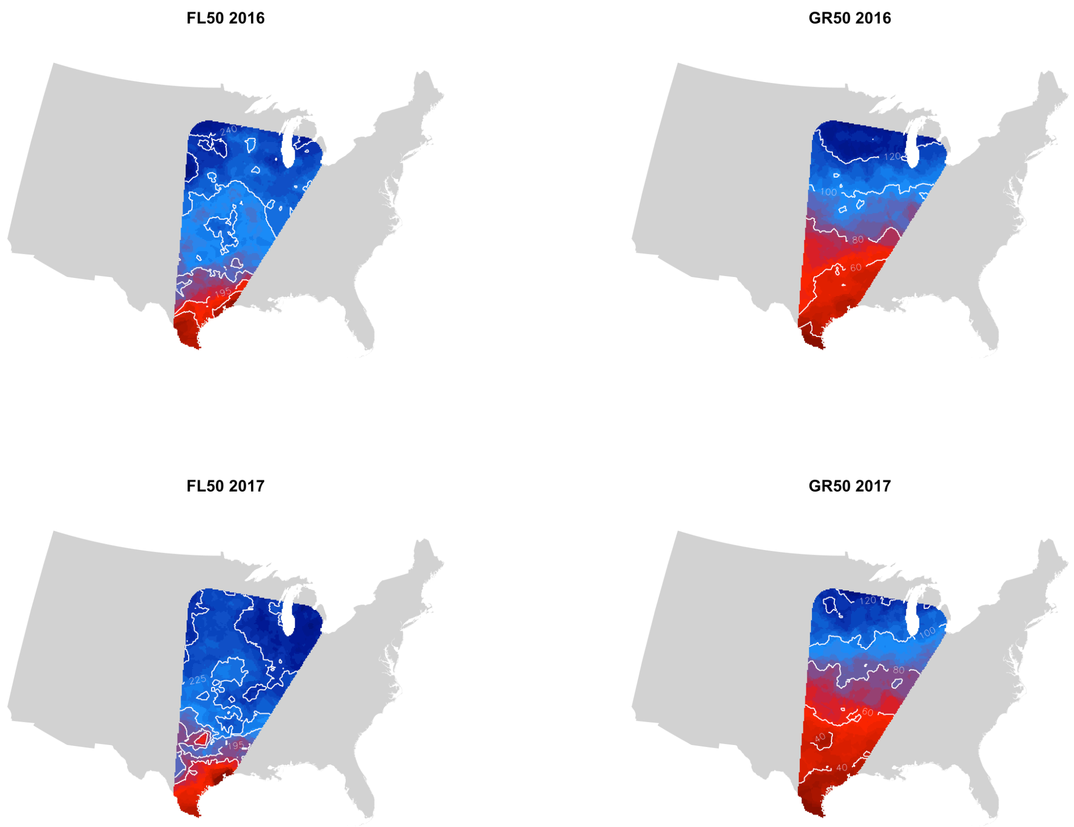

Fig. S16. Prediction of growing season start (left) and end (right) for all 651 NOAA weather stations, interpolated across the landscape. Flowering ("FL50", left) and greenup ("GR50", right) are presented for 2016 (top) and 2017 (bottom). Predicted values are presented along the white contour lines.

Table S1 List of discovered QTL across 2016 and 2017. The position (POS), LOD score, and QTLxE interactions (yes / no) for all significant QTL.

Phenotype	CHR	POS	LOD	QTLxE
BIOMASS	1N	25	6.324	yes
BIOMASS	2K	63	9.841	yes
BIOMASS	3K	66	7.127	yes
BIOMASS	3N	21	7.927	yes
BIOMASS	3N	83	9.427	yes
BIOMASS	4N	33	4.572	yes
BIOMASS	5K	82	6.324	yes
BIOMASS	5N	89	6.057	yes
BIOMASS	6N	44	4.673	yes
BIOMASS	7K	38	4.453	yes
BIOMASS	8K	54	4.021	yes
BIOMASS	9K	22	6.491	yes
BIOMASS	9K	84	6.836	yes
BIOMASS	9N	83	4.032	yes
BIOMASS	9N	119	6.586	yes
FL50	1N	84	5.3	yes
FL50	2K	0	12.472	yes
FL50	2K	60	10.604	yes
FL50	2N	73	22.39	yes
FL50	3K	48	4.928	yes
FL50	3N	40	3.932	yes
FL50	4K	9	15.651	yes
FL50	5K	24	3.843	yes
FL50	5K	89	7.678	yes
FL50	5N	5	43.992	yes
FL50	5N	92	45.577	yes
FL50	6K	51	4.331	yes
FL50	6N	0	5.073	yes
FL50	7K	12	3.575	yes
FL50	8N	13	4.753	no
FL50	9K	13	6.235	yes
FL50	9N	22	4.902	yes
FL50	9N	58	4.65	yes
FL50	9N	111	6.522	yes
GR50	1K	62	5.556	yes
GR50	1N	43	5.696	yes
GR50	1N	80	4.777	yes
GR50	2K	10	7.866	yes
GR50	2K	65	4.051	yes
GR50	2N	99	5.702	yes
GR50	3N	19	3.709	yes
GR50	3N	88	4.162	yes
GR50	5K	89	9.18	yes
GR50	7K	1	5.004	no
GR50	7K	59	5.63	yes
GR50	8K	30	9.036	yes
GR50	9K	57	3.636	yes
GR50	9N	51	4.235	yes
HT	2K	11	8.572	yes
HT	2K	65	7.611	yes
HT	2N	47	10.205	yes
HT	3K	35	14.093	yes
HT	3N	6	5.112	yes
HT	3N	75	11.556	yes
HT	4K	45	3.772	yes
HT	5K	0	7.203	yes
HT	5N	11	5.114	yes
HT	5N	89	14.093	yes
HT	6K	30	4.717	yes
HT	6N	60	7.141	yes
HT	7K	31	4.647	no
HT	9K	24	7.844	yes
HT	9K	81	3.92	no
HT	9N	47	7.977	yes
TC	1N	35	3.741	no
TC	2K	63	11.629	yes
TC	3K	35	8.043	no
TC	3N	11	4.256	no
TC	3N	51	5.925	yes
TC	3N	83	4.362	yes
TC	5K	51	5.733	yes
TC	6K	10	3.58	yes
TC	6N	50	3.783	yes
TC	7K	33	7.7	yes
TC	8N	49	5.238	no
TC	9K	81	4.622	yes
TC	9N	38	7.78	yes
TC	9N	83	8.671	yes

Table S2 Heritability (H^2) by trait and site with standard errors (SE).

SITE	YEAR	TRAIT	H^2	SE
BRKG	2017	GR50	0.31	0.11
BRKG	2017	FL50	0.58	0.09
BRKG	2017	TC_EOS	0.21	0.11
BRKG	2017	HT_EOS	0.66	0.07
BRKG	2017	BIOMASS	0.69	0.07
CLMB	2017	GR50	0.49	0.08
CLMB	2017	FL50	0.63	0.07
CLMB	2017	TC_EOS	0.3	0.1
CLMB	2017	HT_EOS	0.58	0.07
CLMB	2017	BIOMASS	0.6	0.07
KBSM	2017	GR50	0.17	0.1
KBSM	2017	FL50	0.78	0.05
KBSM	2017	TC_EOS	0.46	0.09
KBSM	2017	HT_EOS	0.79	0.05
KBSM	2017	BIOMASS	0.6	0.07
KING	2017	GR50	0.53	0.09
KING	2017	FL50	0.61	0.08
KING	2017	TC_EOS	0.23	0.11
KING	2017	HT_EOS	0.31	0.11
KING	2017	BIOMASS	0.12	0.11
LINC	2017	GR50	0.31	0.11
LINC	2017	FL50	0	0.13
LINC	2017	TC_EOS	0.61	0.08
LINC	2017	HT_EOS	0.62	0.08
LINC	2017	BIOMASS	0.61	0.08
MNHT	2017	GR50	0.56	0.09
MNHT	2017	FL50	0.88	0.04
MNHT	2017	TC_EOS	0.33	0.11
MNHT	2017	HT_EOS	0.82	0.05
MNHT	2017	BIOMASS	0.55	0.09
OVTN	2017	GR50	0.72	0.06
OVTN	2017	FL50	0.55	0.09
OVTN	2017	TC_EOS	0.61	0.08
OVTN	2017	HT_EOS	0.61	0.08
OVTN	2017	BIOMASS	0.71	0.07
PKLE	2017	GR50	0.65	0.07
PKLE	2017	FL50	0.79	0.05
PKLE	2017	TC_EOS	NA	NA
PKLE	2017	HT_EOS	0.62	0.07
PKLE	2017	BIOMASS	0.46	0.09
STIL	2017	GR50	0.53	0.09
STIL	2017	FL50	0.73	0.06
STIL	2017	TC_EOS	0.34	0.11
STIL	2017	HT_EOS	0.7	0.07
STIL	2017	BIOMASS	0.67	0.07
TMPL	2017	GR50	0.8	0.05
TMPL	2017	FL50	0.53	0.09
TMPL	2017	TC_EOS	0.45	0.1
TMPL	2017	HT_EOS	0.59	0.08
TMPL	2017	BIOMASS	0.63	0.08

Table S3. Location and timing of growing season by site. Greenup and flowering are given as Julian Days.

id	lat	lon	First Greenup	Last Flowering
BRKG	44.3068	-96.6705	122	240
CLMB	38.8969	-92.2178	90	230
KBSM	42.41962	-85.37127	113	258
KING	27.54986	-97.88101	23	133
LINC	41.1543	-96.4153	109	256
MNHT	39.1407	-96.6389	80	231
OVTN	32.3029	-94.9794	47	197
PKLE	30.38398	-97.72938	39	163
STIL	35.99115	-97.04649	69	220
TMPL	31.04338	-97.3495	52	215

Table S4. Bioclim variables at each site. Columns 1-19 indicate the numbers of each of the 19 bioclim variables.

id	1	2	3	4	5	6	7	8	9	10	11	12	13	14	15	16	17	18	19
BRKG	61	126	27	11695	287	-179	466	182	-99	205	-99	584	99	10	60	257	35	254	35
CLMB	122	117	30	9515	314	-75	389	219	-6	241	-6	977	121	38	30	326	139	294	139
KBSM	87	113	29	9696	286	-100	386	198	-30	208	-43	906	98	39	24	275	147	271	153
KING	223	117	41	5686	354	75	279	267	159	291	145	685	119	24	46	266	103	201	110
LINC	102	127	29	10550	312	-117	429	212	-40	234	-40	772	111	16	52	308	58	295	58
MNHT	123	131	31	10029	329	-90	419	225	-12	249	-12	852	136	20	51	352	70	322	70
OVTN	181	128	37	7359	344	6	338	220	267	272	81	1132	121	54	18	331	220	225	277
PKLE	195	122	38	6920	351	32	319	233	118	281	103	835	113	42	32	278	160	185	163
STIL	155	131	33	8915	346	-43	389	203	36	267	36	881	135	29	40	317	108	250	108
TMPL	190	126	36	7408	355	13	342	231	107	281	89	872	115	47	26	284	177	186	179

Table S5. Growing season-adjusted climate variables. Variables with '14' in the name represent the most extreme 14-day period for that value. For example, HOT14 is the mean TMAX of the hottest 14-day period.

id	season	TMAX	TMIN	PRCP	HOT14	COLD14	DRY14	WET14	CV_PRCP	CV_TMAX
BRKG	early	28.5	-1.06	2.46	23.65	0.79	0.42	6.39	1.24	1.32
CLMB	early	28.29	-2.21	4.74	23.75	1.09	0.8	13.44	1.26	1.29
KBSM	early	27.45	-0.27	2.72	23.19	4.08	0.71	4.36	1.02	0.99
KING	early	34.34	0.98	0.5	28.8	9.04	0.04	1.43	1.34	0.74
LINC	early	30.23	-1.11	4.35	25.99	2.7	0.65	11.94	1.27	1.15
MNHT	early	27.05	-5.05	3.57	23.89	-1.15	0.04	11.38	1.41	1.56
OVTN	early	28.73	1.43	2.78	26.94	7.26	0.03	5.48	1.4	0.81
PKLE	early	29.7	4.41	2.01	26.21	8.67	0	4.55	1.41	0.71
STIL	early	28.91	-2.58	3.12	20.19	3.17	0	7.25	1.41	1.03
TMPL	early	28.98	4.27	5.43	27.23	9.48	0.24	17.56	1.38	0.68
BRKG	middle	32.22	7.22	2.49	30.08	10.95	0.05	7.66	1.39	0.66
CLMB	middle	32.39	6.29	3.33	30.14	10.04	0.13	5.41	1.35	0.71
KBSM	middle	31.42	9	1.59	29.08	10.65	0.14	4.57	1.33	0.66
KING	middle	33.5	7.76	2.3	31.09	12.52	0	7.93	1.41	0.6
LINC	middle	35	7.72	2.44	32.91	10.99	0	5.42	1.41	0.71
MNHT	middle	35.24	4.42	2.9	33.86	6.47	0.16	6.1	1.34	0.96
OVTN	middle	30.75	7.5	4.68	28.33	10.65	0.92	10.54	1.19	0.64
PKLE	middle	32.41	9.39	1.29	30.5	14.19	0	3.54	1.41	0.52
STIL	middle	34.07	5.56	4.49	33.23	7.24	0.44	12.99	1.32	0.91
TMPL	middle	32.65	9.36	4.66	31.51	12.62	0.1	16.29	1.4	0.61
BRKG	late	30.67	8.33	4.14	28.69	12.74	0.49	7.78	1.25	0.54
CLMB	late	33.54	12.02	3.4	32.7	15.33	0.64	5.84	1.14	0.51
KBSM	late	33.36	5.67	1.22	29.65	7.68	0.29	3.29	1.19	0.83
KING	late	36.66	9.38	0.83	33.04	15.44	0	2.07	1.41	0.51
LINC	late	34.67	-17.78	2.19	28.35	4.27	0	4.81	1.41	1.04
MNHT	late	38.45	12.2	3.86	36.44	15.79	0	9.42	1.41	0.56
OVTN	late	34.88	15.32	4.01	33.99	17.88	0.38	11.01	1.32	0.44
PKLE	late	36.17	13.65	2.82	34.96	17	0	6.88	1.41	0.49
STIL	late	37.86	17.8	4.51	36.34	19.38	0	9.36	1.41	0.43
TMPL	late	37.09	20.29	1.12	36.54	21.87	0.02	3.39	1.4	0.36

Table S6. PCA-prediction models for each QTL. P-values, estimates, and standard errors (se) are from the meta-regression model.

qtl	pca	pvalue	estimate	se
1K@62_AxB_GR50	PC2	1.69E-06	-0.1529598	0.03195185
1N@43_CxD_GR50	PC1	4.14E-12	0.10650203	0.01536345
2K@0_AxB_FL50	PC5	2.49E-10	-0.9511095	0.15031327
2K@60_CxD_FL50	PC1	1.16E-07	0.21816976	0.04116291
2K@65_CxD_GR50	PC1	2.58E-11	0.09875858	0.0148087
2K@65_CxD_HT	PC1	7.48E-04	0.25569738	0.07584762
2N@73_AxB_FL50	PC4	1.84E-03	-0.4084895	0.13114279
3K@35_CxD_HT	PC3	1.74E-03	-0.3631476	0.1159534
3N@19_AxB_GR50	PC1	6.03E-05	-0.0580793	0.01447824
3N@83_AxB_TC	PC1	1.93E-03	-1.5832242	0.51054438
3N@83_CxD_BIOMASS	PC1	1.10E-14	0.01720839	0.00222714
4K@9_AxB_FL50	PC3	8.82E-09	-0.3846621	0.06687543
5K@0_AxB_HT	PC3	1.88E-03	0.36690346	0.1180204
5K@24_AxB_FL50	PC4	1.02E-05	-0.5815387	0.13180912
5K@89_AxB_GR50	PC1	4.78E-07	0.07165651	0.01423217
5K@89_CxD_FL50	PC1	7.91E-05	0.15998541	0.04053088
5K@89_CxD_GR50	PC1	3.40E-20	0.12896932	0.01401011
5N@11_AxB_HT	PC1	6.24E-05	0.28829614	0.07201102
5N@5_AxB_FL50	PC1	7.22E-16	0.34054802	0.04221671
5N@89_AxB_BIOMASS	PC4	1.22E-04	-0.026266	0.00683545
5N@89_AxB_HT	PC3	8.13E-05	-0.4543317	0.1152933
5N@92_AxB_FL50	PC4	2.07E-29	-1.4905572	0.13237842
6K@30_CxD_HT	PC1	2.13E-03	-0.2229823	0.072604
6N@60_AxB_HT	PC1	1.08E-04	-0.2831396	0.07313074
7K@59_AxB_GR50	PC1	6.33E-12	0.09706265	0.01412441
8K@30_CxD_GR50	PC1	8.22E-07	0.06991322	0.01418117
9K@13_AxB_FL50	PC5	9.68E-04	-0.5173235	0.15677964
9K@22_AxB_BIOMASS	PC4	1.51E-04	-0.026314	0.00694317
9K@24_AxB_HT	PC4	1.09E-04	-0.8319401	0.21500469
9K@57_AxB_GR50	PC1	4.07E-04	-0.0506913	0.01433696
9N@111_CxD_FL50	PC1	1.85E-04	0.15666809	0.04190389
9N@51_AxB_GR50	PC1	2.28E-11	0.09444649	0.01412441
9N@83_CxD_BIOMASS	PC1	3.08E-11	-0.015284	0.00230084
9N@83_CxD_TC	PC1	4.00E-07	-2.4772993	0.48873342

Table S7. Model parameter summaries for estimation of first green-up and last flowering at each site across two years.

Trait	Coefficient	T-value	P-value
Green-up	Year	-12.179	3.53e-09
	PCA1	-50.884	< 2e-16
	PCA9	4.552	0.000381
	PCA10	-2.018	0.061854
Flowering	PCA1	8.503	4.03e-07
	PCA3	-2.342	0.03342
	PCA4	3.913	0.00139
	Year*PC6	2.950	-0.00993
